# Dealing with suicidal patients – a challenging task: a qualitative study of young physicians' experiences

**DOI:** 10.1186/1472-6920-6-44

**Published:** 2006-08-23

**Authors:** Tordis Sørensen Høifødt, Anne-Grethe Talseth

**Affiliations:** 1 Department of Clinical Psychiatry, Division of Clinical Medicine, University of Tromsø/Department of Psychiatric Research and Development, University Hospital of Northern Norway, Åsgård, N-9291 Tromsø, Norway; 2 Tromsø College, Faculty of Health Sciences, M.H. bygget, Breivika, 9293 Tromsø, Norway

## Abstract

**Background:**

Suicide is a major public health problem and treating suicidal patients represents one of the most challenging and complex clinical situations for young physicians. Education of physicians is considered an important strategy in suicide prevention. Young physicians often meet suicidal patients early in their career. Limited information is available about how newly educated physicians experience treating suicidal patients. The aim of the study was to shed light on the meaning of newly educated physicians' lived experiences in treating patients at risk of committing suicide.

**Methods:**

Thirteen newly educated physicians narrated their experiences with suicidal patients. The interview text was transcribed and interpreted using a phenomenological-hermeneutical method inspired by Ricoeur's philosophy.

**Results:**

Three main themes and ten themes were noted: ***Striving for relatedness***: relating with the patient; not being able to relate with the patient; ***Intervening competently***: having adequate professional knowledge; performing professionally; having professional values; evaluating one's own competence; and ***Being emotionally involved***: accepting one's own vulnerability; feeling morally indignant; feeling powerless and accepting one's own fallibility. The recently educated physicians clearly described the variety of emotional and ethical dilemmas that arose in meeting suicidal patients and the professional challenge facing this clinical situation. The findings were interpreted in the perspective of communication, clinical decision-making and attention to the professional's emotional reactions.

**Conclusion:**

An examination of the experiences of young physicians treating suicidal patients reveals three main themes that were a professional challenge for them: Striving for relatedness, Intervening competently and Being emotionally involved. Support for young practitioners that are treating these patients is likely important both to facilitate learning and also for their own well-being. This increased understanding can open up for the patient's suffering and affirm the patient's sense of life. The study provides additional background for educators designing training programs for physicians who will be treating suicidal patients.

## Background

Dealing with suicidal patients represents one of the most challenging clinical situations for young physicians. Suicide is a major public health problem. It is among the top three causes of death in the population aged 15–34 years worldwide [1]. In Norway the number of suicides increased from 8 to 16 per 100000 mean population from 1970 to 1988 [2]. A National Plan for Suicide Prevention was instituted in 1994. The Norwegian plan for suicide prevention primarily focused on measures aimed at enhancing programs for particular risk groups. Central measures to attain this aim included acquiring new knowledge through research, distributing knowledge through education and information activities, and giving advice and counseling to professional groups working on clinical and preventive projects [3]. In 2004 the suicide rate was 12 per 100000 mean population [2].

Suicide is a complex phenomenon and difficult to predict. However, at least some and perhaps many suicidal acts might be prevented if suicidal patients receive the necessary support and help [4,5]. Studies indicate that about 45% of those who commit suicide had contact with primary care providers within the last month preceding the suicide [6]. One-third of those who completed suicide had contact with mental health services within the year of their death, while more than 75% had contact with primary care providers [6]. Education of physicians in depression recognition and treatment has been a documented intervention to reduce suicide rates [7].

Wolk-Wassermann [8,9] studied how staff in intensive care units experienced meeting suicidal patients using exploratory non-directive and semi-structured interviews. Suicidal patients evoked positive feelings such as empathy and sympathy, and negative feelings such as distancing, aggression, guilt, anxiety and fear. In a study based on narrative interview data, Talseth et al [10] emphasized the importance of physicians confronting their own feelings of mortality, vulnerability and fallibility in order to improve their interactions with suicidal patients [10].

Therapists' reactions to patients who commit suicide have been studied by using semi-structured questionnaires combined with written narratives. These studies show that clinicians express shock, guilt, fear of blame, self-doubt, anger and betrayal [11,12] – similar to the reactions among close family members of patients committing suicide [12]. Gitlin [13] described the reactions to patients' suicide as the most psychologically difficult experiences encountered in the life of a clinician. In a study of how medical students evaluated their encounter with suicidal patients [14], the students expressed how difficult they felt confronting suicidal behaviour and the great responsibility physicians are given in this situation. Young physicians often meet suicidal patients early in their career. Negative experiences with these frequently complex and critical situations might impact their professional confidence and represent obstacles for further learning and development [15]. Physicians entering practice have found these situations both difficult and inspiring, and may often need supervision and support [16].

It was not possible to locate studies encompassing how newly educated physicians experienced treating suicidal patients. This study used in-depth interviews to obtain a deeper understanding of these experiences. The focus here was upon the subjective experiences of the physicians. In this study the focus is not on the patients, considering received treatment and satisfaction.

### The aim of the study

The aim of the study was to shed light on the meaning of newly educated physicians' lived experiences in treating patients at risk of committing suicide.

## Methods

### Design

This study used a qualitative design, using a phenomenological-hermeneutical method.

### Subjects

The informants had been medical students graduating in 2000 at the University of Tromsø, Norway. In Norway an 18-month internship, including internal medicine, surgery and primary health care, follows graduation from medical school and precedes full authorization as a medical practitioner. The informants had completed their internship during the last six months prior to the interview. As this study was part of a project on the development of psychiatric competence among medical students, an invitation to participate in interviews, together with questionnaires for addressing other aspects of the project, was sent to 54 students. Forty-four responded (81%) anonymously to the questionnaires and 16 of these agreed to participate in in-depth interviews. The intention was to interview all 16, but three could not be interviewed for practical reasons. The informants were seven men and six women; all about 30 years of age. The interviewees were not different from all those eligible for participation in gender and age. At the time of the interview nine of the 13 informants were working in general practice, the remaining in hospitals, but none of them in psychiatric services.

### Ethics

Informants received written information about the project and gave informed consent. They were guaranteed confidentiality and anonymity in the publications of the results. The Regional Ethics Committee and the Norwegian Data Protection Agency approved the study in 2002.

### The interviews

The first author conducted the narrative interviews [17] during the first six months of 2002. They lasted 90 to 120 minutes and were audio-taped and transcribed verbatim.

The informants were invited to narrate a story from treating suicidal patients in their own practice. The narrated stories are mostly taken from the context of a primary physician's practice, but some of the informants told about patients they had treated in a hospital setting.

The interviewer used clarifying questions and invited the informants to elaborate sequences that were unclear, by asking: What did you think? What did you feel? What did you do? The questions were intended to help the informants to continue their account, which in turn led to a new phase of questions. The purpose of the narrations was to make the experiences explicit and through interpretation, provide meaning to what was talked about.

### Data analysis

The hermeneutic approach presupposes that the researchers' interpretation and understanding is based on their pre-understanding [18,19]. The unconscious part of the researcher's pre-understanding consists of parts of his or her own culture, language, history, which though taken for granted, still influences the interpretation of the text [19]. This subjectivity can be counteracted by application of a strict method of interpretation [20].

The narrative interviews have been interpreted using the phenomenological-hermeneutic approach described by Ricoeur [20,21]. It has previously been applied in a similar way [22,23]. This method focuses upon understanding and interpreting the meaning of phenomena of life experiences. Ricoeur [20,21] describes interpretation of a text as a dialectic process from understanding to explanation and then from explanation to critical comprehension. The interpretation includes three phases. The first phase, the naive understanding, includes several readings of the text in an open-minded manner trying to gain an initial overall grasp of the meaning of the text as a whole. The second phase, structural analysis, is directed towards the structure of the text. The text was divided into "meaning units", sentences or whole paragraphs that are related by their content. The content of each meaning unit was condensed, abstracted and organised into sub themes, themes and main themes. In the third phase, comprehensive understanding, the text was considered as a whole while taking into account the first reading, the structured analysis, the research questions, literature and the researchers' pre-understanding.

A critical comprehension was then formulated and reflected upon.

## Results

### Naive understanding

Treating suicidal patients represented difficult clinical situations and required considerable emotional effort on the part of the physicians. Physicians often worried about practical matters before meeting the patients. They frequently feared that the patient would commit suicide before they arrived. They were concerned about establishing a good relationship with the patient, doing a careful risk assessment and then taking appropriate action. They felt great responsibility for the patient's life and feared making a wrong decision. Involuntary hospital admissions were difficult decisions to them as it meant restricting the patients' autonomy.

Physicians frequently reported a variety of emotional reactions encountering suicidal patients, such as anxiety, pain, grief, but also aggression. Some patients activated irritation and helplessness in the physicians; especially those who repeatedly threatened to hurt themselves. The physicians had often been thinking through how they might react if their patient committed suicide. Following a suicide, the reactions were strong; although only a few of the informants had experienced that situation. Many of the young physicians reported experiences related to suicidal behaviour in their own lives and considered these as useful in treating these patients.

### Structural analysis

The structural analysis involved the explanation of the meaning of treating suicidal patients narrated by the physicians, and seventeen sub themes, ten themes and three main themes were developed. The main themes, themes and sub themes are presented below:

#### 1. Striving for relatedness

##### A. Relating with the patient

###### Establishing a relationship

The informants worried about not having enough time to establish a positive relationship with suicidal patients. They explained the purpose of their visit and wanted to understand the patients' problems and the reasons they were suicidal. A part of relating was to decide where to sit in the room and how to emotionally meet a desperate and crying patient. One stated:

*It is difficult to know where to start when the person only sits there and cries... I did not feel that it was right to hold around her. She sat in the chair by herself and seemed to want to sit there. So I sat down in the sofa besides, together with the cats*.

###### Trusting the patient

When the physicians had a feeling of mutual understanding and trust with the patient, they relied on the patient's statements. The doctors described that knowing the patient over time made it easier to sort out what to do. One informant said:

*When I feel a good connection, I think that the patient is being honest and is telling me as much as possible. When the patient finishes his story and one can reply, then you could perhaps get eye contact saying "I can understand what you mean", you receive this glimpse, and a little pause, and then you go on talking*.

The relationship between the physician and the patient might fluctuate during the same consultation, from one of mutuality to one in which there were difficulties in making a connection. In these situations the patients had often used alcohol or drugs. One informant said:

*Things changed between periods where we had good contact, managed to move further and got the patient a little off her thoughts of suicide. However, again and again, she returned to saying that she had nothing to live for*.

##### B. Not being able to relate with the patient

###### Not having emotional relationship

Sometimes physicians did not manage to establish a solid dialogue and were insecure about how the patient was feeling. However, with the patients who showed signs of serious mental illness, the doctors knew what to do and found the situation easier to handle. They also described situations when communication was superficial, yet they sensed that the patients had problems they did not want to talk about. A few informants had experienced that such patients had had serious suicidal plans and committed suicide shortly after the consultation. One of the informants recalled such a situation:

*I tried to see if I could get through to him some way... We talked a little about school, what he had done the last days, but I could not find an opening. When I asked whether he had some problems, he would not go into them*.

###### Mistrusting the patient

Sometimes doctors were in doubt if they could rely on the patient and if the threats of suicide were real or only a means to get attention. They might have met the patient before and felt fooled or manipulated, often along with a plea for addictive medication. One physician said:

*He had used addicting medication. He was appealing to get them, it was obvious that he did not want to kill himself, but he wanted other things*.

#### 2. Intervening competently

##### A. Possessing adequate professional knowledge

###### Collecting information

Physicians asked the patients about their suicidal thoughts, plans for committing suicide, current life situation, recent losses and network. They registered if and how the patients talked about their future and tried to assess the degree of impulse control and unusual behaviour. One informant said:

*I try to get an impression of the social situation, friends, family, job, how he feels in his relationship to these people and his life situation, how he looks upon his future and perhaps the past, experiences of his daily life, his life situation, how he thinks about himself and his relations to these different elements in his life situation*.

###### Making clinical observations

Physicians described the patients' clothes, hygiene, smells, bruises or other sign of self-harm and emotions such as crying, despair or toughness, signs of alcohol or drugs and registered the patient's sense of reality. One informant stated that:

*He was so serious all the time. You could see that he was striving, he couldn't loosen up, and he carried a burden with him all the time. He ate well, slept well, was well kept, but his [pent up] emotions dominated the situation*.

###### Making a diagnostic formulation

Physicians included a diagnostic formulation as part of the assessment of suicidal risk. One interviewee described a situation as follows:

*He had both a personality disorder, unstable and perhaps he was also psychotic. He also had an alcohol addiction, but not to narcotics*.

##### B. Performing professionally

###### Having expectations of oneself and from others

The physicians wanted to be helpful and useful. They felt that the patient's family or friends and other health care workers also expected them to be available, competent and able to make the necessary decisions. One informant said:

*It is my attitude as a physician to do something, I want to help, treat in one way or other*.

Another stated:

*There were expectations especially from young nurses. They thought we knew a lot*.

###### Focusing on results

Even before meeting the patient, the doctors went mentally through how to arrange transportation and procedures for admission to hospital. They wanted their meeting with the patient to result in specific actions, such as follow up with the family physician, home care and psychiatric services, or admittance to a hospital. One physician said:

*As long as I know that a plan has been established, it may be an admission to hospital, as an emergency or the next day or in some other way of following the patient up... that one has made some decision and provided a guarantee for the patient*.

##### C. Having professional values

###### Having responsibility for the patient's life

Physicians felt a heavy responsibility for saving the patient's life. One informant expressed:

*I was both scared and felt helpless because I could not do anything either way in the situation, but on the other hand, I was involved and had a responsibility*.

Often the situation was unclear as to whether the patient was able to be responsible for her/himself.

###### Being in an ethical dilemma

The informants described the dilemma they felt when admitting a patient to hospital against his or her own will. On the one hand it provided the necessary treatment and safe care, but on the other hand it curtailed the patient's autonomy. Even if medical and legal criteria were met, the doctors felt this as an infringement upon the patient's autonomy. They described great distress in communicating this decision to the patients. One informant related that:

I did not feel bad in making the decision, but I felt uncomfortable watching the reactions of the patient... I have gone over it again and again, is the decision justifiable, have I tried out everything else possible?

##### D. Evaluating one's own competence

###### Concern about one's own reputation

The physicians were sensitive to comments and critique. The doctors feared losing control and tried to keep up a professional appearance. They were anxious that they might make the wrong assessment when the consequences could be very serious. Fear of being accused of making a faulty decision was expressed by one as follows:

*Did I do something wrong – that was the immediate reaction – am I going to be charged? The consequences: I was afraid of the consequences; maybe I have made an error, made the wrong assessment*.

#### 3. Being emotionally involved

##### A. Accepting one's own vulnerability

###### Facing the suffering of the patient

Confronting the forces of life and death activated the young physicians' anxiety and pain. One informant stated:

*One gets scared of the forces expressed in "I want to commit suicide". You are supposed to be professional, on the other hand one also has personal feelings, which patients like these, really can stir. One might get upset, feel pain and fear*.

###### Opening up for one's own experiences about suicide

The physicians shared personal stories related to suicide that were reactivated during the meeting with the suicidal patient. They viewed those experiences as useful as it allowed them to better relate to and understand their patients. One physician stated:

*It was a very close experience, but I do not think it has made things more difficult for me, rather somehow easier*.

###### Sorrow and guilt in the aftermath of a suicide

Some of the doctors had experienced that one of their patients committed suicide. The physicians felt grief. At the same time they questioned whether they had made a wrong decision. One interviewee said:

The personal experience was grief, I had talked to him just a few days before this happened, so it was close. I reacted immediately with a sense of fright, what have I done?

###### Meeting the bereaved in the aftermath of a suicide

Some had gone to a family in shock and despair and felt helpless. One informant related that:

*I met the parents and it was really hard. I felt helpless and did not know what to say. Perhaps one does not always have to say so much. I got the feeling that I wanted to say or do something. It was very difficult that situation. It is one of the most uncomfortable situations I have ever been in*.

##### B. Feeling morally indignant

Sometimes the physicians experienced aggression and annoyance towards suicidal patients, especially if the situation also represented a danger to other people. One expressed:

*When people do things like that, I get angry; I try to keep that to myself, because it is not very useful in the relationship with the patient. You get angry with a person because he just drives into an oncoming trailer. He didn't really have huge problems. His action was indefensible*.

##### C. Feeling powerless

Doctors felt helpless and tired of the patients who repeatedly made suicide attempts. One informant said:

*It just gets to be too many of them, you get so tired. "The intoxes" with those pills are often people that come again and again... It sounds awful, but I sometimes thought, if they really want to kill themselves, why don't they take a large enough dose [to do the job]*.

##### D. Accepting one's own fallibility

The doctors reported that they had to live with the uncertainty that one of their patients might commit suicide. They had thought about how they would react and had tried to prepare themselves for the day when they would lose a patient. One interviewee said:

*How good are we at picking up those who are really at risk to commit suicide? There is no guarantee that a patient will survive to the next day even if he has been to the doctor*.

## Discussion

The perspective of the interviews was broad, encompassing the physicians' thoughts, clinical observations, considerations, actions and own emotional reactions. Three main themes appeared to cover the newly educated physicians' experiences treating suicidal patients: Striving for relatedness, Intervening competently and Being emotionally involved. There were no apparent thematic differences arising from physician gender or the context in which the narrated story took place (primary care or a hospital setting).

Establishing contact and communication is recognised as an essential aspect in preventing suicide [10]. Communication includes both professional knowledge and empathy. It requires interplay of feelings, rationality and ethical considerations, as well as taking into account the patient's history, the actual situation and a perspective for the immediate future [24]. When these physicians experienced mutual trust with and an understanding of the patient, they felt comfortable in making assessments and treatment plans. In relationships where this was not the case the physicians experienced difficulties along with feelings of helplessness, insecurity and/or anger. However, if the patient showed signs of serious mental illness, their path forward was clearer and the situation was easier to deal with. These findings are in accordance with those of Wolk-Wassermann [8]. It is interesting that when a suicidal state could be attributed to a serious mental disorder, the suicidal crisis was then objectified and something instrumental could be done. It was as if the physician was then able to gain a more detached perspective and to distance him/herself from the difficult human interactional aspects of the situation. It seemed to relieve him/her from the feelings of inadequacy.

The young physicians also described aggression and aversion facing suicidal patients. Such feelings might influence their ability to deal with the situation in an adequate way [9,25]. Maltzberger [25] discussed the intense negative emotions that interacting with suicidal patients can evoke in psychotherapists. He found the concept of counter-transference useful in understanding and sorting out which reactions are basically related to the patient, and what has to do with the physician her/himself.

The young physicians wanted their meetings with suicidal patients to result in a specific action, but described the dilemma of feeling paternalistic and the fear of allowing more patient autonomy than might be prudent [26]. The doctors found these decisions both difficult and burdensome. Patients both seek an emotional signal of understanding and ways to proceed [27]. To be able to affirm the patient and focus adequately on a proper course of action could be a professional challenge.

The physicians had to deal with their own feelings that arose in the contact with suicidal patients. Physicians' own pain, anger and annoyance arose when facing self-destructive behaviour. Sadness and guilt were predominating in the aftermath of a suicide. The reactions of these newly educated physicians corresponded well with other descriptions of therapists' reactions to suicidal patients [8,11,25]. Those studies have generally focused upon therapists with considerable experience. These reactions appear as basically human and can arise in any role or stage of one's career and even in the first encounter with a suicidal patient.

The young physicians communicated their fears of making mistakes, the stress of their uncertainties and concern about their reputation. The informants also shared their personal stories related to suicidal behaviour and seemed to accept both their own vulnerability and fallibility. Emotionally taxing work has been studied in relation to burn-out and job satisfaction. Both positive (personal achievement and job satisfaction) and negative (emotional exhaustion and burn-out) consequences have been noted [28]. Making the first years of medical practice meaningful, both from a learning perspective and as a personal and professional development, is challenging [16]. These newly educated physicians clearly described the many dilemmas and challenging emotions that arose in treating suicidal patients. Thus it would seem prudent to provide some form of supervision and/or support from experienced colleagues. This could serve to promote both learning and the younger physician's sense of well-being. This increased understanding can open up for the patient's suffering and affirm the patient's sense of life.

Education of physicians is considered a key strategy in suicide prevention [7]. This study provides additional information about the subjective experiences of young physicians facing suicidal patients. Hopefully it will be of assistance to educators who are developing clinical teaching programs that address dealing with suicidal patients.

### Methodological considerations

Those who accepted to be interviewed did not differ in age and gender from others in the group of newly educated physicians. However, they could well represent a group of doctors that are more interested, reflective or, perhaps, more personally involved in the issues surrounding suicide. However, their reports seemed to represent experiences typical for young physicians. They were also congruent with findings from previous research, which lends them some additional credibility [29]. The informants talked about situations in the past and reconstructed what happened. The narration of personal experiences has been found to be a useful method previously [30]. In addition, both authors have analyzed and interpreted the data. Nonetheless, the text could well have multiple meanings and be interpreted in various ways. The present interpretation seems credible, yet remains but one of several possibilities [20]. Phenomenological-hermeneutic interpretation should not be seen as factual knowledge, but rather be regarded as contributing to the discourse about the topic at hand [31].

The strength of this study is the thorough examination of in-depth interviews that addressed both the behavioural, cognitive and emotional aspects of the young physicians' experiences. The considerable and consistent detail in the data also adds to the conclusion that there is high content validity in the material [32]. The narratives illustrated the complex interplay between cognition, emotion and action [33].

The interviewer is a psychiatrist and was open to emotional expression during the interview, and the informants seemed to use this opportunity to talk about their own emotional reactions. The interviewer's knowledge of the field provided a necessary prerequisite for understanding the narratives of the newly educated physicians. By consciously taking into account the pre-understanding of the authors/researchers in the research process, it is generally accepted that critical reflection remains possible [19]. Given the results and the considerations above, this study would appear to have credibility (transferability) beyond the sample in the given setting.

Several interviewees expressed that the interview had been personally valuable for them. One's own narrative can provide both a healing effect and new insights. These would seem to be some of the benefits of participating in research interviews. [34].

## Conclusion

An examination of the experiences of young physicians treating suicidal patients reveals three main themes: Striving for relatedness, Intervening competently and Being emotionally involved. These three tasks were a professional challenge for them. Support for young practitioners that are treating these patients is likely important both to facilitate learning and also for their well-being. This increased understanding can open up for the patient's suffering and affirm the patient's sense of life.

The study provides additional background for educators designing training programs for physicians who will be treating suicidal patients.

## Competing interests

The author(s) declare that they have no competing interests.

## Authors' contributions

TSH has conceived the study, carried out the interviews, done the analysis and written the draft of the manuscript. AGT has contributed substantially in the process of design, analysis and has critically revised the manuscript. Both authors have read the manuscript and approved it for publication (Vancouver Convention).

## Pre-publication history

The pre-publication history for this paper can be accessed here:


